# *Ab initio* Study of Anchoring Groups for CuGaO_2_ Delafossite-Based p-Type Dye Sensitized Solar Cells

**DOI:** 10.3389/fchem.2019.00158

**Published:** 2019-03-29

**Authors:** Ana B. Muñoz-García, Laura Caputo, Eduardo Schiavo, Carmen Baiano, Pasqualino Maddalena, Michele Pavone

**Affiliations:** ^1^Department of Physics “Ettore Pancini”, University of Naples Federico II, Comp. Univ. Monte Sant'Angelo, Naples, Italy; ^2^Department of Chemical Sciences, University of Naples “Federico II”, Comp. Univ. Monte Sant'Angelo, Naples, Italy

**Keywords:** density functional theory, p-type DSSCs, anchoring groups, Cu delafossites, delafossite surface

## Abstract

Here we report the first theoretical characterization of the interface between the CuGaO_2_ delafossite oxide and the carboxylic (–COOH) and phosphonic acid (–PO_3_H_2_) anchoring groups. The promising use of delafossites as effective alternative to nickel oxide in p-type DSSC is still limited by practical difficulties in sensitizing the delafossite surface. Thus, this work provides atomistic insights on the structure and energetics of all the possible interactions between the anchoring functional groups and the CuGaO_2_ surface species, including the effects of the Mg doping and of the solvent medium. Our results highlight the presence of a strong selectivity toward the monodentate binding mode on surface Ga atoms for both the carboxylic and phosphonic acid groups. Since the binding modes have a strong influence on the hole injection thermodynamics, these findings have direct implications for further development of delafossite based p-type DSSCs.

## Introduction

The increasing world energy demands have boosted research toward the development of technologies that can exploit renewable sources in an efficient way (Hagfeldt et al., 2010). Being inexhaustible and relatively well spread all over the globe, solar energy has the highest potential to satisfy the present and future global energy needs. The photovoltaic (PV) field has rapidly evolved from traditional and commonly used silicon panels based on semiconductor p/n junctions to competitive thin film technologies relying on less abundant elements (Parida et al., 2011). Branching off from these devices, dye-sensitized solar cells (DSSC) arose 20 years ago, initially as cost-effective alternatives to solid-state photovoltaic solar cells. Now that the cost competitiveness with Si is less relevant, DSSCs are still in the spotlight, due to their lightweight, transparency, flexibility and best performance at diffuse and low-intensity light, which make them suitable for portable and indoor applications (Benesperi et al., 2018).

The most studied DSSCs are photoanodes where the electric current arises from the electron injection from the LUMO of a photoexcited dye to the conduction band of the n-type semiconductor (typically TiO_2_ or ZnO) where such dye is anchored. To date, traditional n-type DSSCs with metallic counterelectrodes have reached ~14% in photo–conversion efficiencies (PCE), while more recently the closely related hybrid organic/inorganic perovskite-based set-ups have exceeded the ~20% PCE (Yang et al., 2017). In order to overcome the intrinsic efficiency drawbacks of first generation DSSCs with only one chromophore, tandem cells have been proposed (He et al., 2000; Green, 2003; Nakasa et al., 2005; Nattestad et al., 2010). In tandem cells, a n-DSSC is coupled to a photocathode made of a p-type semiconductor that is sensitized by a dye with a red-shifted adsorption (with respect to dye at the photoanode), so that a larger portion of the solar spectrum can be harvested. Besides the potential higher photocurrent and PCEs for PV applications, tandem cells can be devised as well as photoelectrochemical devices able to perform photoinduced chemical reactions of great relevance in the energy conversion scenario (e.g., water splitting) (Prévot and Sivula, 2013). The actual application of tandem cells is however hampered for the limited efficiencies of the p-type DSSCs, which reach efficiencies of ~2%, still far below the ~14% of their n-type counterparts (Odobel et al., 2010).

A main culprit behind such poor performances has been ascribed to NiO, which is the most used p-type semiconductor (SC) in p-DSSCs due to its low cost and easy manipulation (Morandeira et al., 2005; Mori et al., 2008; Odobel et al., 2012). Unfortunately, NiO presents substantial intrinsic drawbacks: low electrical conductivity, low hole mobility and high valence band edge potential with respect to the most common I^−^/${\text{I}}_{3}^{-}$ electrolyte, which results in a too low photocathode open circuit potential (V_OC_) (Odobel and Pellegrin, 2013). The development of new efficient p-DSSC as alternative to NiO is thus of primary relevance.

Copper delafossites with formula CuMO_2_ (M = Al, Ga, Cr…) (Sullivan et al., 2016) have emerged among the few materials that can indeed outperform NiO as p-SC. Delafossites have wide optical bandgap and low valence band edge (Marquardt et al., 2006; Yu et al., 2012; Kumar et al., 2013). Nattestad et al. reported one of the first consistent comparisons between NiO and CuAlO_2_ in p-DSSC, obtaining V_OC_ values of 218 and 333 mV, respectively (Nattestad et al., 2011). Later, experimental and theoretical investigations confirmed the lower VB_edge_ position of CuAlO_2_ compared with NiO (Yu et al., 2014; Das et al., 2015; Schiavo et al., 2018). CuGaO_2_ and CuCrO_2_ were also reported as possible alternative to NiO, with lower band edge positions and higher transparency (Yu et al., 2012). With respect to NiO-based p-DSSC, with the new Ga and Cr delafossite oxides the measured increase in V_OC_ was about 160 and 110 mV, respectively (Renaud et al., 2012; Powar et al., 2014). Several works show that the efficiency of these materials in p-DSSCs can be further improved by doping them with a divalent cation (e.g., Mg) at the M site (Scanlon and Watson, 2011; Jiang et al., 2013). This, in fact, enhances the p-type conductivity and has a positive effect on the morphology of the nanoparticles, which can expose a higher surface area resulting in a better light harvesting (Renaud et al., 2014).

However, despite the positive effect on V_OC_, the replacement of NiO with CuMO_2_ does not always result in an overall significant increase of photocurrent and/or PCE. While the V_OC_ is determined by the p-SC VB_edge_ vs. the electrolyte redox couple reduction potential, the overall cell efficiency strongly depends also on the interfacial electronic processes that occur between the semiconductor and the sensitizer. First, the lower the position of the valence band, the smaller is the driving force for hole injection to a given dye. For this reason, typical “p-type dyes” used for NiO may not be suitable for delafossites. For example, Renaud et al. tested the C343 prototype coumarin dye on CuGaO_2_ and did not measure any photocurrent (Renaud et al., 2012). P1, PMI-6T-TPA, or PMI-NDI dyes are used to sensitize CuGaO_2_, CuAlO_2_, and CuCrO_2_ delafossites, delivering small but non-zero current (Yu et al., 2012, 2014).

Another critic issue related to the delafossite-dye interface is the limited light-harvesting arising from poor dye coverage (Renaud et al., 2014). When synthesized via conventional solid-state reaction, CuMO_2_ tend to form large-size (>1 μn) anisotropic plate-like particles that densely stack along the basal planes, resulting in a limited surface area available for dye sensitization (Yu et al., 2014). With focused but less straightforward synthetic methods (e.g., following the hydrothermal route) it is possible to obtain smaller particles, close to the ideal 20–40 nm (i.e., the typical TiO_2_ nanoparticle size in n-DSSC) (Xiong et al., 2013; Yu et al., 2014). Nevertheless, these alternative synthetic routes are challenging, also depending on the element at the M site, and advances toward increasing dye coverage are highly desirable, even for particles with non-ideal shape or morphology.

In this context, an open issue is whether the dye-anchoring groups that are used for binding the dye to the rocksalt NiO surfaces are also good for a stable and irreversible binding to the Cu-based delafossite oxide most exposed surfaces. To address this specific issue related to the dye-electrode interface in delafossite-based p-DSSCs, we present here a first-principles study on two anchoring groups on CuGaO_2_ (001) surface: carboxylic acid (–COOH), which is the most common anchoring group used in both n- and p-type DSSCs, and phosphonic acid (–PO_3_H_2_), which is one of the anchoring groups explored in NiO-based p-type DSSCs in search for higher efficiencies (Pellegrin et al., 2011; Klein et al., 2018) and guarantees more stability in aqueous environments where COOH tends to desorb from oxide surfaces (De Angelis et al., 2011; Galliano et al., 2017).

First-principles calculations are a valuable tool for obtaining an atomistic insight on the structures, chemical interactions, and electronic features at the dye-electrode interfaces, thus providing a better understanding of the undergoing elementary processes. While electronic, adsorption and excited properties and level alignment of dyes anchored on n-type semiconductors (mainly TiO_2_) have been studied extensively using density functional theory (DFT) and time-dependent DFT (Martsinovich and Troisi, 2011; Labat et al., 2012; Adamo and Jaquemin, 2013; Bai et al., 2014), p-type semiconductor/dye interfaces have been investigated only more recently, and, in particular, considering NiO as semiconductor. An early work by Preat et al. focuses on computing the electronic excitation and electron injection processes of P1 and derived dyes using as model for NiO a single Ni atom capped by a hydroxyl group to ensure the overall neutrality of the system (Preat et al., 2011). Regarding periodic-slab and spin-polarized DFT calculations, taking into account also the intrinsic magnetism of NiO, only few recent computational studies have addressed the interaction of dye/anchoring groups with the most stable surface of NiO, i.e., the (001) (Muñoz-García and Pavone, 2015; Kontkanen et al., 2016; Wykes et al., 2016; Carella et al., 2018). Wykes et al. (2016) have studied formic and benzoic acid (HCOOH and PhCOOH) as prototypes of the most commonly used carboxyl-based anchoring groups, as well as mono and dimer phenyl-silane [PhSi(OH)_3_] anchoring candidates, due to the known capacity of alkoxysilanes to form Si-O-metal bonds. Besides different adsorption strengths, they find that such anchoring groups induce different shifts of the NiO VB and CB edges. A previous work by our group on the coumarin dye C343 (which features –COOH as anchoring group) and its analogous with –PO_3_H_2_ adsorbed on NiO (001) has shown that not only the nature of the binding group but also the binding modes largely affect the thermodynamic driving force for hole injection, due to the interface dipole generated by the H released to the surface in bi- and tri-dentate cases (Muñoz-García and Pavone, 2015). Piccinin et al. recently reported *ab initio* molecular dynamics simulations on the coumarin dye with –PO_3_H_2_ anchoring group on NiO (001) including explicit water molecules, showing also that the hole injection driving force (NiO-VB/Dye-HOMO level alignment) is very sensitive of the specific anchoring modes and their dynamics in a protic solvent (Piccinin et al., 2017). These works highlight the key role played by the anchoring ligand adsorbed on the surface on the overall performance of the DSSC.

While a few theoretical works have addressed the study of the electronic structure of bulk Cu-based delafossites for DSSC applications (Gillen and Robertson, 2011; Schiavo et al., 2018), the features of the dye-delafossite interface are essentially unexplored. In this work, we perform state-of-the-art DFT-based periodic calculations on anchoring groups –COOH and –PO_3_H_2_ on delafossite CuGaO_2_, which has been proposed as the most convenient for DSSC applications thanks to its wide band gap and easier p-type doping with respect to other delafossites such as CuAlO_2_ or CuCrO_2_ (Schiavo et al., 2018). After evaluating the change in the VB edge position upon Mg doping, we report adsorption energies and relevant structural and electronic features of the different anchoring groups/modes on the surface, taking into account the different combination of surface metal sites (Cu or Ga) that can be involved in the dye anchoring. Finally, we present a comparison of our results on CuGaO_2_ with those on NiO.

## Computational details and structural models

We performed periodic spin-polarized density functional theory (DFT) calculations with projector-augmented wave (PAW) potentials (Blöchl, 1994; Kresse and Joubert, 1999) and plane waves (PW) basis set by using the Vienna *Ab Initio* Simulation Package (VASP, version 5.4.1) (Kresse and Hafner, 1993, 1994; Kresse and Furthmüller, 1996a,b). The generalized-gradient approximation of Perdew Burke and Ernzherof (PBE) has been exploited for the exchange-correlation density functional (Perdew et al., 1996, 1997). To describe the strong-correlated nature of Cu *d* electrons we applied the rotationally invariant DFT+U approach of Dudarev (Dudarev et al., 1998) as implemented in VASP (Rohrbach et al., 2003). As in previous works on Cu(I)-containing oxides, we applied an average U-J effective value of 6 eV on Cu *d* electrons (Isseroff and Carter, 2012; D'Arienzo et al., 2017; Schiavo et al., 2018). SCF energy convergence threshold was set to 10^−5^ eV, and for minimum-energy structural optimizations the total forces on each atom were set to be all below 0.05 eV·Å^−1^. Regarding numerical parameters, with a kinetic energy cut-off of 750 eV for the PW and a k-point Monkhorst-Pack (Monkhorst and Pack, 1976) sampling grid of 6 × 6 × 2 for bulk CuGaO_2_ (see below) we obtained convergence of total energies within 5 meV per formula unit. K-point sampling for the slabs used have been scaled accordingly.

**Figure 1:**
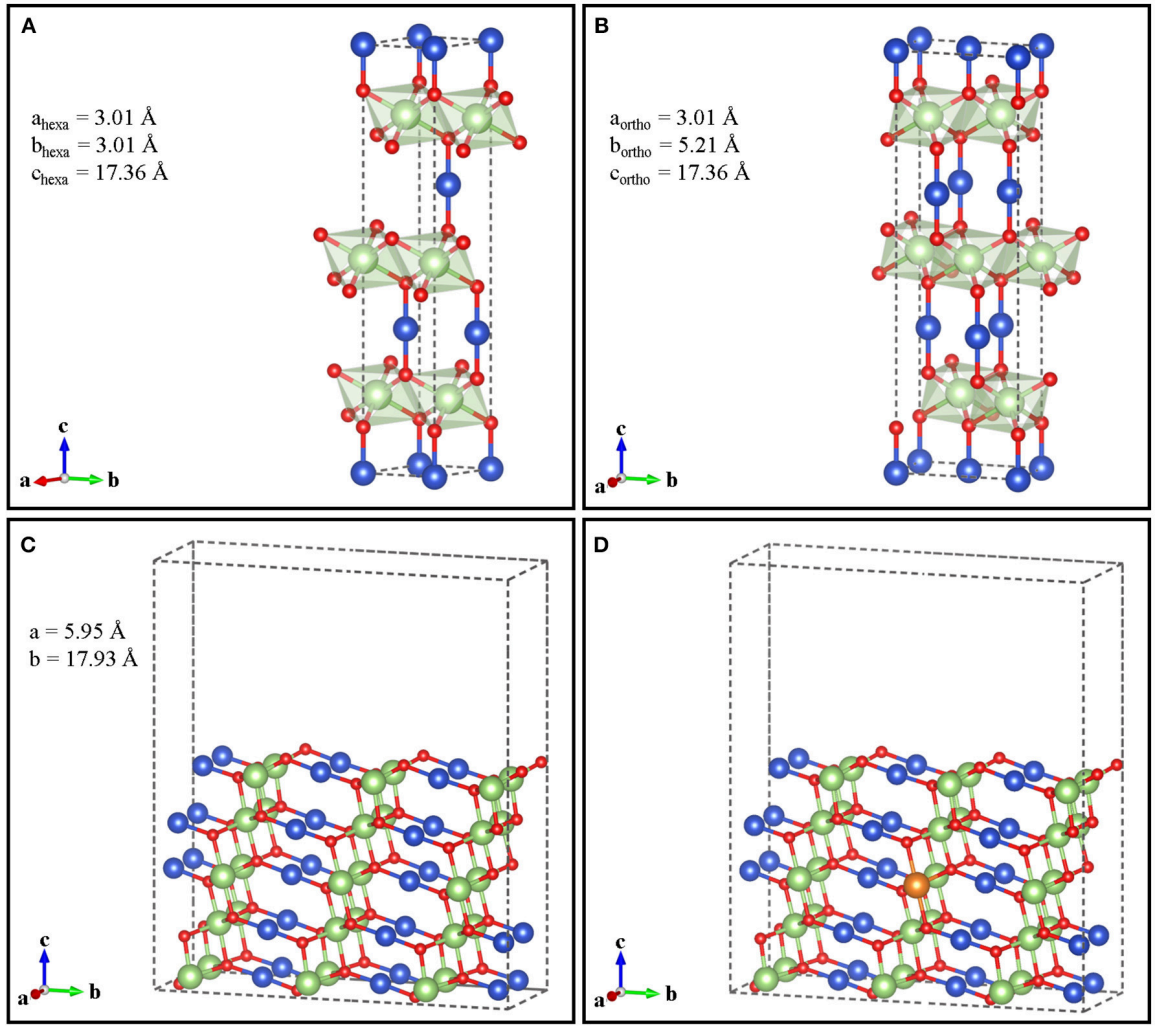
Hexagonal **(A)** and orthorhombic **(B)** unit cells of bulk CuGaO_2_ with calculated cell parameters. Undoped **(C)** and Mg-doped **(D)** (011) orthorhombic surface. Color code: Cu (blue), Ga (green), O (red), and Mg (orange).

Although delafossites can present different polytypes (Marquardt et al., 2006), here we have considered the 3R phase, which is the only one detected with X-ray diffraction in CuGaO_2_ (Renaud et al., 2014). The hexagonal 3R polytype of CuGaO_2_ structure has the Ga(III) ions accommodated in a distorted octahedral cavity in the oxygen sublattice, generating layers of Ga(III)O_6_ units sharing their edges. These layers are separated by Cu(I) ions, linked in a linear geometry with two oxygens in two different layers (Figure 1A). For simplicity, we have built an orthorhombic supercell with a_ortho_ = a_hexa_, b_ortho_ = √3 b_hexa_, and c_ortho_ = c_hexa_ (Figure 1B). For surface calculations, we considered the (011) surface (012 in the hexagonal system), which has been identified among the preferred facets of delafossite crystals (Das et al., 2015; Alkhayatt et al., 2016) and, in particular, has the lowest surface energy in CuGaO_2_ (Schiavo et al., 2018). We cleaved such surface from orthorhombic bulk CuGaO_2_ with the theoretically determined equilibrium lattice constants (a = b = 2.98 Å, c = 17.64 Å), which deviate <3% from the experimental values (a = b = 2.97 Å, c = 17.17Å) (Ishiguro et al., 1981; Köhler and Jansen, 1986; Crottaz et al., 1996) For electronic structure calculations, the (011) surface was represented as slab with a (2 × 1) periodicity in the xy plane, five tri-atom layers of thickness and 10.5 Å of vacuum, in order to avoid images interaction along the *z* direction (Figure 1C). For Mg-doped CuGaO_2_, we have placed one Mg substituting one Ga atom on the central atom layer (Figure 1D), as it has been proven that the Mg substitution occurs at the Ga site (Jiang et al., 2013). This delivers a Mg-doped slab with 3.3% content of Mg (all % signs throughout the text are intended as atom %). An additional slab with (1 × 1) lateral periodicity has been used to study Mg-doped CuGaO_2_ surface with 6.7% of dopant, by substituting with Mg a single Ga atom in the central slab layer. Both in the undoped and Mg-doped cases, atomic positions of all the atoms have been allowed to relax.

The relaxed CuGaO_2_ surface slab was used to compute the workfunction and vacuum energy level in order to determine the VB edge absolute position (i.e., with respect to the NHE) using the approach proposed by Toroker et al. (2011):

$$\begin{array}{l} {\text{VB}}_{\text{edge}}=\text{BGC}-{\text{E}}_{\text{vac}}-½{\text{E}}_{\text{g}} \end{array}$$

Where BGC is the band-gap center of the slab from our PBE+U calculations and E_g_ is the eigenvalue gap of CuGaO_2_ bulk calculated at the HSE level of theory, i.e., 2.1 eV (Schiavo et al., 2018), which is very close to the value of 2.2 eV reported by Iozzi et al. (2015). E_vac_ is the vacuum energy evaluated from the electrostatic potential along the direction normal to the surface plane. For the Mg-doped system, where the BGC is ill-defined due to the typical shoulder of empty valence band states above the Fermi level that appears in the density of states (DOS) in p-type semiconductors, we have calculated the VB_edge_ from the VB_edge_ of un-doped CuGaO_2_ applying a shift computed from the difference of the workfunctions of the two systems (WF_Mg:CuGaO2_-WF_CuGaO2_).

To survey the adsorption modes and the corresponding adsorption energies/properties of –COOH and –PO_3_H_2_ anchoring groups on CuGaO_2_ (001), we have chosen as capping substituent the simple methyl group (–CH_3_). This is a safe approximation for studying anchoring properties since, as we show below, they depend very little on the dye skeleton.

CH_3_-COOH and CH_3_-PO_3_H_2_ molecules have been placed in one side of CuGaO_2_ (001) slab with 2 × 1 of lateral periodicity and 3 tri-atom layers of thickness. Adsorption energy vary of <50 meV by increasing the lateral periodicity to 3 × 1 and of <25 meV by increasing the slab thickness to 5 tri-layers. Vacuum was increased to 15 Å to prevent interaction between the slab periodic images. Since molecules are adsorbed onto only one side of the slab, dipole corrections have been applied to avoid long-range polarization from the periodic images along the *z* direction (Neugebauer and Scheffler, 1992). We have considered all the possible anchoring modes for each group, taking also into account the different surface sites where the anchoring can be bound. Final geometries of these structures have been obtained relaxing all atomic coordinate of the molecule and of the two uppermost CuGaO_2_ tri-atom layers and by freezing those of the bottommost layers.

After relaxation, we have calculated adsorption energies as follows:

$$\begin{array}{l} \Delta {\text{E}}_{\text{ads}}={\text{E}}_{\text{slab}+\text{CH}3-\text{X}}-{\text{E}}_{\text{slab}}-{\text{E}}_{\text{CH}3}-\text{X} \end{array}$$

from the energies of the total system (E_slab+CH3−X_), the relaxed pristine slab (E_slab_) and the isolated molecule (E_CH3−X_), with X = CO_2_H or PO_3_H_2_. Using the relaxed geometries from gas-phase calculations, we have computed also the adsorption energies in acetonitrile, a common solvent used in p-DSSCs, by performing single-point energy calculations with the implicit solvent scheme implemented in VASP (Mathew et al., 2014) with relative dielectric constant ε = 35.688.

For comparison with NiO, we have studied CH_3_COOH and CH_3_PO_3_H_2_ molecules anchored on NiO (001) following an analogous procedure. Structural and computational details for NiO (001) surface slab (kinetic energy cut-off, k-point sampling, U-J values, slab thickness, lateral periodicity) are exactly those exploited in a recent previous work on the C343/NiO (001) interface (Muñoz-García and Pavone, 2015).

## Results and discussion

**Figure 2:**
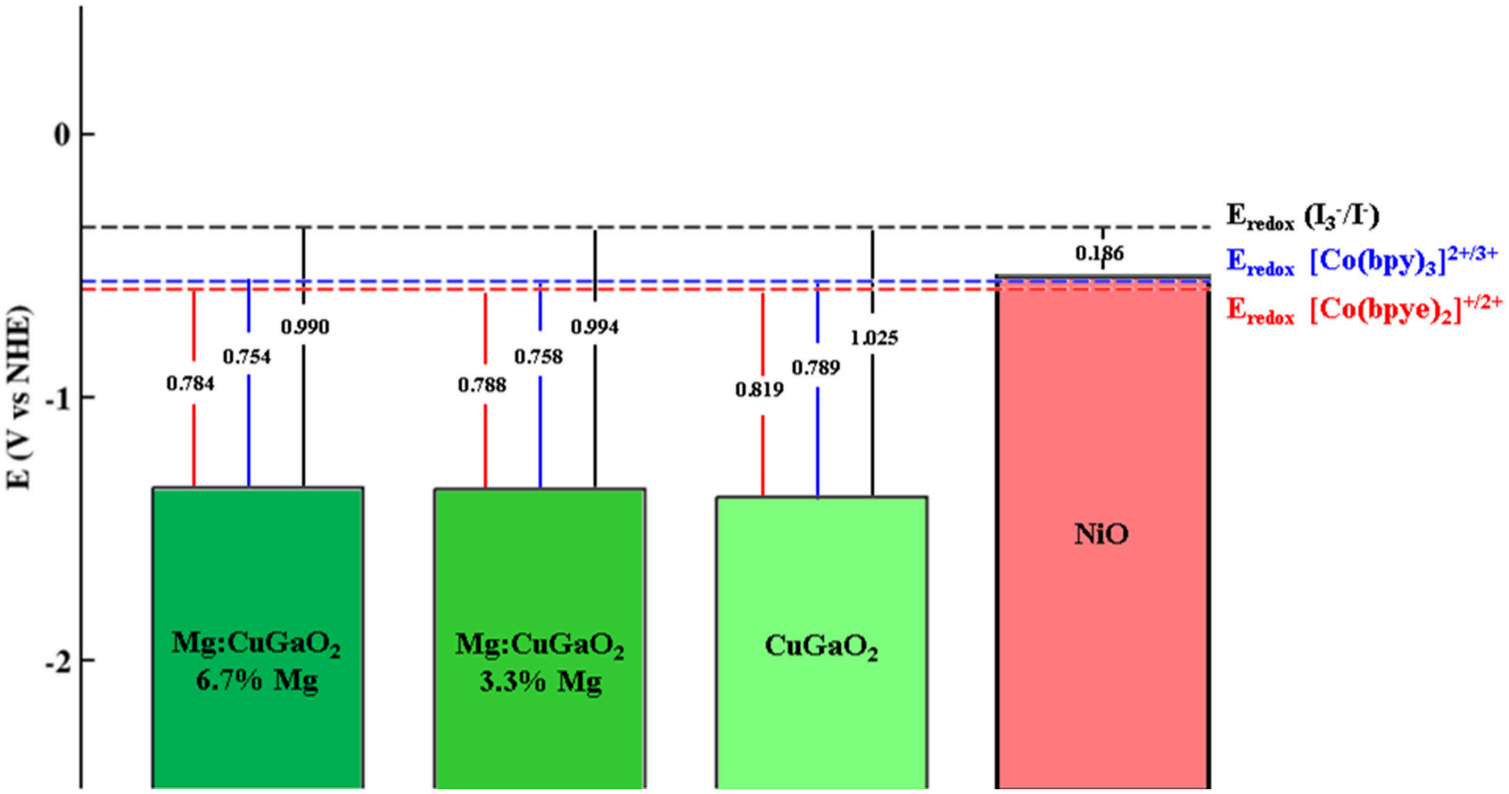
Calculated absolute positions of the valence band edges for, from left to right: Mg:CuGaO_2_ with two different dopant concentrations (6.7 and 3.3%), undoped CuGaO_2_ and NiO. Dashed lines represent the redox potentials of the three electrolytes taken from experimental studies: ${\text{I}}_{3}^{-}$/I^−^ (Boschloo and Hagfeldt, 2009), [Co(bpy)_3_]^2+/3+^ (Cong et al., 2016), and [Cu(bpye)_2_]^+/2+^ (Feldt et al., 2010). For completeness open circuit voltage calculated with respect to those electrolytes are reported.

In p-type DSSCs, a first key parameter to assess the suitability of a p-semiconductor is the absolute position of its VB edge with respect to the electrolyte redox potential: the difference between these two values determines the open circuit potential V_OC_ of the cell (Qin et al., 2008; Preat et al., 2011). For Mg doped CuGaO_2_, Renaud et al. (2014) have reported that Mg doping has a positive effect on the photoelectrode by increasing the specific surface area (SSA) of CuGaO_2_, but only CuGaO_2_ nanoparticles with low concentration of Mg (1%) deliver higher efficiencies than pristine CuGaO_2_. Higher concentrations of Mg (up to 5%), which increases further the SSA, do not lead to higher photocurrent. This drawback has been ascribed to the formation of structural defects or electronic features that promote undesired electron-hole recombination processes. Regarding the V_OC_ with respect to the tris(4,4′-bis-tert-butyl-2,2′-bipyridine)cobalt(II/III) redox couple, the same authors (Renaud et al., 2012) measured a decrease in V_OC_ values at increasing Mg contents. To understand this behavior, we computed VB edge positions of Mg-containing CuGaO_2_ in comparison to that of pristine CuGaO_2_ so to dissect to what extent Mg doping affects the V_OC_ without any other structural defects. We have evaluated two different Mg concentrations, 3.3 and 6.7%, by substituting one Ga from the central tri-layer of the slab in the 5-tri-layer slabs with (2 × 1) and (1 × 1) periodicity, respectively. Figure 2 shows the resulting band edge potentials vs. NHE of CuGaO_2_, Mg-doped CuGaO_2_ and NiO together with common electrolytes employed in dye sensitized solar cells. All the reduction potentials of the redox mediators are measured in an acetonitrile solution, we refer the interested reader to the experimental references (Boschloo and Hagfeldt, 2009; Feldt et al., 2010; Cong et al., 2016) for the exact composition of the electrolyte.

The VB edge of CuGaO_2_ with 3.3% Mg is of 29 meV higher in energy with respect to the undoped case, and it rises up of another 4 meV when increasing Mg concentration from 3.3 to 6.7%. These numbers are of the same order of magnitude of the experimental values reported by Renaud et al. where a decrease of V_OC_ of 30 meV for 1% Mg with respect to the undoped case and a further decrease of 10 meV when doping is increased up to 5% Mg (Renaud et al., 2014). Such little increase of the VB edge by Mg doping is still very low in comparison how low VB edge is in CuGaO_2_ with respect to NiO, which makes both CuGaO_2_ and Mg-doped CuGaO_2_ suitable for p-DSSCs not only with ${\text{I}}_{3}^{-}$/I^−^ electrolyte (Boschloo and Hagfeldt, 2009) but also with other redox couples that are not compatible with NiO, such as cobalt(II/III) tris(2,2′-bipyridine) [Co(bpy)_3_]^2+/3+^ and copper(I/II) bis(1,1-bis(2-pyridyl)ethane) [Cu(bpye)_2_]^+/2+^ (Feldt et al., 2010; Cong et al., 2016). It is important to note that these results provide only a qualitative picture of the energy level alignment between the electrode and the electrolyte redox couple, many features are missing in our model (solvent, band bending at the interface, effects of ionic species, etc.). However, the computed trend is consistent with the V_OC_ values observed in experiments and this agreement provides a good assessment on the quality of our surface slab model.

Understanding how the dye anchoring groups interact with the delafossite surface is of primary relevance: high adsorption strengths are needed to provide high dye coverages and, thus, high photoconversion efficiency. Moreover, in the case of H-containing anchoring groups, the individuation of preferred anchoring modes can help to explain and predict the emerging efficiencies, as different anchoring modes of the same anchoring group can deliver different band alignments between the dye HOMO and the p-semiconductor VB edge (Muñoz-García and Pavone, 2015; Zhang and Cole, 2015; Adineh et al., 2016).

We have surveyed all possible anchoring modes for both –COOH and –PO_3_H_2_ functional groups on CuGaO_2_ (011), taking into account the different possible surface adsorption sites and combinations of them. Thus, we have considered monodentate binding on either Ga or Cu surface atoms (M-Ga and M-Cu, respectively), bidentate binding on Ga/Ga, Cu/Cu or Ga/Cu surface atom pairs (B-Ga-Ga, B-Cu-Cu, and B-Ga-Cu) and, for CH_3_-PO_3_H_2_ we have considered also the two possible tridentate binding modes, on two Ga atoms plus one Cu atom or on two Cu atoms plus one Ga atom (T-Ga-Ga-Cu or T-Cu-Cu-Ga). For M cases, we have added the sub index “H” when the OH group forms an H bond to an oxygen surface atom upon relaxation. In B and T cases, the H atoms have been attached to surface oxygen atoms, as far as possible from the anchoring group to avoid artificial overstabilization and distortion of the structures due to H-bond formation between the released H and the O atoms from the anchoring molecules.

**Figure 3:**
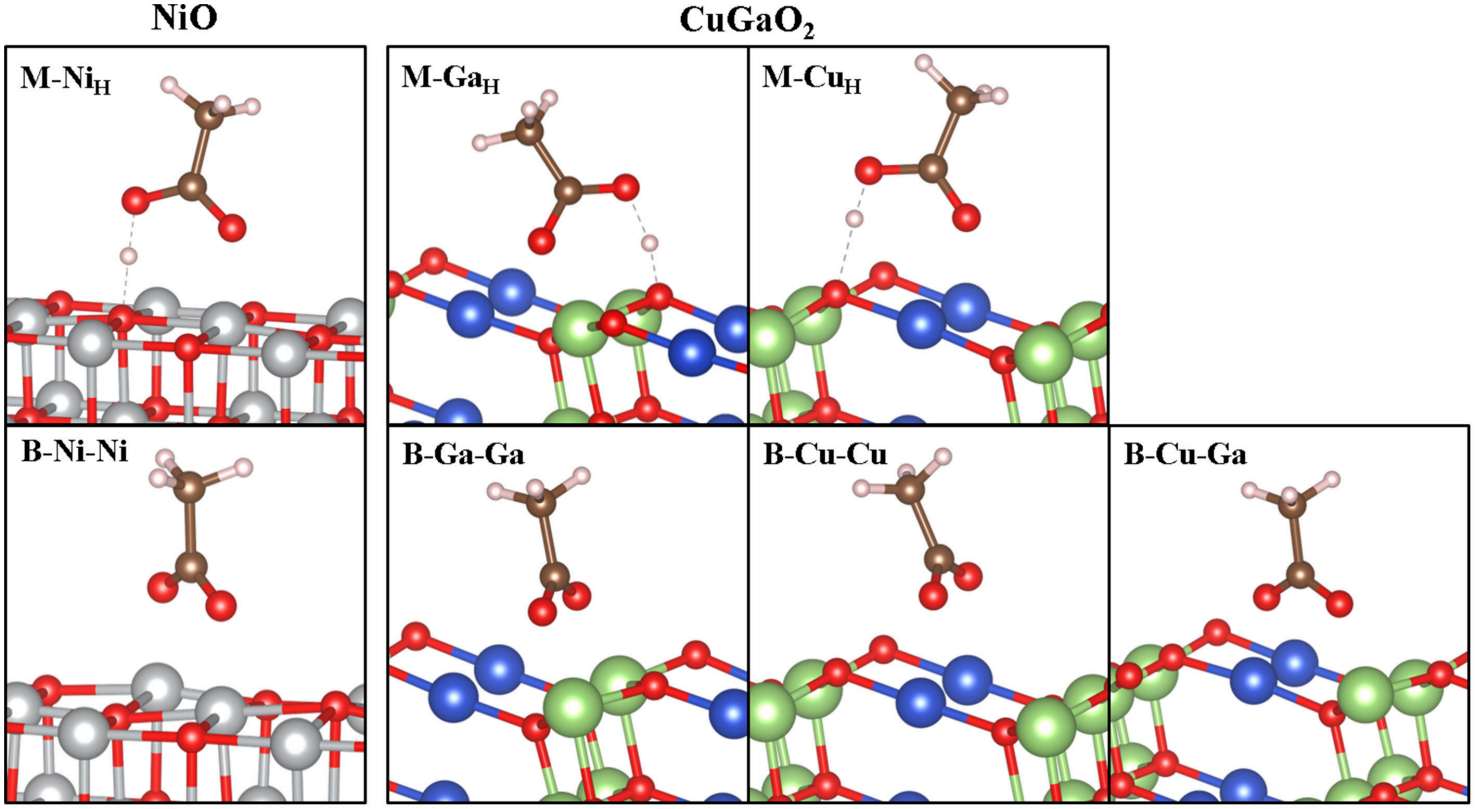
Optimized structures of CH_3_COOH on CuGaO_2_ (011) 2 × 1 × 3 L slab (right) and on NiO (001) (left). Labels according to the anchoring mode [monodentate (M) and bidentate (B)] and to the surface atoms involved in the adsorption process. Sub index “H” indicates H bonding (dashed gray line) between the OH group and surface oxygen atom upon relaxation in M cases. Color legend: Ni (gray), Cu (blue), Ga (green), O (red), C (brown), and H (light pink).

Relaxed geometries of CH_3_COOH on CuGaO_2_ (011) in monodentate (M) and bidentate (B) binding modes are shown in Figure 3 and corresponding selected structural parameters together with binding energies (E_ads_) are listed in Table 1. xyz files of the intermediates structures are reported in Supplementary Information. These geometries and E_ads_ are compared to those of CH_3_COOH on NiO (001).

**Table 1:** Selected structural parameters for relaxed CH_3_COOH anchored on CuGaO_2_ (011) and on NiO (001) together with those of the isolated molecule calculated at the DFT-PBE+U level of theory in vacuum.

		**CH**_**3**_**COOH—Main structural parameters** **(Å)**								
	**Anchoring mode**	**d_**O1-C**_**	**d_**O2-C**_**	**d_**O-H**_**						**E_**ads**_ (eV)**
Isolated		1.22	1.36	0.98						
On NiO(001)					**d**_**O1-Ni**_		**d**_**O2-Ni**_		**d**_**H-Os**_	
	M-Ni_H_	1.26	1.38	1.15	2.05		2.05		1.31	−0.81
	B-Ni-Ni	1.27	1.29	–	2.01		2.01		–	−0.88
On CuGaO_2_(011)					**d**_**O1-Ga**_	**d**_**O1-Cu**_	**d**_**O2-Ga**_	**d**_**O2-Cu**_	**d**_**H-Os**_	
	M-Ga_H_	1.28	1.38	1.29	2.03	–	–	–	1.56	−0.76
	M-Cu_H_	1.24	1.33	1.03	–	2.38	–	–	1.60	−0.19
	B-Ga-Ga	1.28	1.28	–	2.03	–	2.05	–	–	0.52
	B-Cu-Cu	1.25	1.27	–	–	1.93	–	1.94	–	1.60
	B-Ga-Cu	1.26	1.28	–	2.03	–	–	1.95	–	0.72

*Labels of anchoring modes as in Figure 3. O_1_/O_2_ correspond to carbonyl/OH oxygen atoms in isolated CH_3_COOH, respectively. O_s_ identifies a superficial oxygen atom. Adsorption energies (E_ads_) in eV are collected in the last column. Bond lengths in the adsorbate (dX-C) and distance between the surface and the adsorbate (dCu-X, dGa-X, dNi-X, dOs-X) with X = O1, O2 or H*.

Adsorbed CH_3_-COOH in M binding present similar structural features in CuGaO_2_ (011) and NiO (001), with the molecule not suffering any significant distortion from the isolated minimum. The main difference resides in the different M-carbonyl oxygen distance to the electrode surface (**d**_**O1−X**_in Table 1), which is much shorter for bonding to a surface Ga (2.03 Å) than to a surface Cu (2.38 Å). The Ga-O_1_ distance in CuGaO_2_ is also very similar to the Ni-O_1_ distance in NiO (2.05 Å) and both values are similar to typical Ga-O_lattice_ and Ni-O_lattice_ in parent solids (2.00 and 2.11 Å in CuGaO_2_ and NiO, respectively). Contrarily, Cu-O_1_ distance is significantly longer than Cu-O_lattice_ distances in CuGaO_2_ (1.87 Å). In CuGaO_2_, our calculations predict slightly longer H bonds between the OH and surface oxygen atoms (**d**_**H−Os**_in Table 1) in CuGaO_2_ with respect to NiO. In bidentate modes, the two oxygen atoms of CH_3_COOH molecule become equivalent and, accordingly, we obtain two equal M-O distances for B-Ga-Ga and B-Cu-Cu cases of CuGaO_2_ and B-Ni-Ni of NiO. Differently for the M case, here the Cu-O_1_ distances are much smaller and similar to those of CuGaO_2_ bulk. In the mixed B-Ga-Cu case, each Ga-O and Cu-O distance is equal to those in B-Ga-Ga and B-Cu-Cu. In spite of the different surface patterns of CuGaO_2_ (011) and NiO (001), M-M distances are very similar in both solids, i.e., d(Ni-Ni)_NiO_ = 2.99 Å and d(Ga-Ga)_CuGaO2_ = d(Cu-Cu)_CuGaO2_ = 2.98 Å, hence the similarities in the binding geometries. Only a slight tilting of the C-C bond with respect to the *z* axis is predicted for B-Ga-Ga and in B-Cu-Cu in CuGaO_2_, having the CuGaO_2_ (011) surface a non-planar pattern.

In spite of these structural analogies between CuGaO_2_ and NiO, the binding energies (E_ads_ in Table 1) of the different anchoring modes of CH_3_COOH to the two surfaces present substantial differences. In NiO, E_ads_ of both M and B modes are negative and close, being E_ads_(B) 0.07 eV more stable than E_ads_(M). We must note that the binding energies reported here for CH_3_-COOH (as well as those indicated below for CH_3_-PO_3_H_2_) on NiO are similar to those considering the full C343 dye (Muñoz-García and Pavone, 2015) within 0.1 eV, and deliver the same result of B being more stable than M [E_ads_(M)-E_ads_(B) = 0.12 eV for C343]. Thus, we can consider the CH_3_- capping group as a good approximation for studying adsorption properties of different anchoring groups on the electrode surfaces. Such negative and similar E_ads_ values can be translated into expecting NiO covered by the dye in the two anchoring modes in approximately equal ratio in experimental conditions. In CuGaO_2_, instead, there is a strong preference for the monodentate binding because all B modes surveyed present a positive (i.e., not favorable) E_ads_. In particular, M binding is stronger at the Ga site than at the Cu site, with E_ads_ values similar to those for NiO. This is consistent with the trend of transition metal-oxo complex bond dissociation energies (BDE) (Luo, 2007) or Ni-O and Ga-O (−366 and −374 KJ/mol), with a smaller value for Cu-O BDE (−287 kJ/mol). Moreover, while surface Ni/Ga atoms are unsaturated species in NiO (001)/CuGaO_2_ (011), surface Cu retains its bulk-like linear coordination with two oxygen atoms in CuGaO_2_ (011).

**Figure 4:**
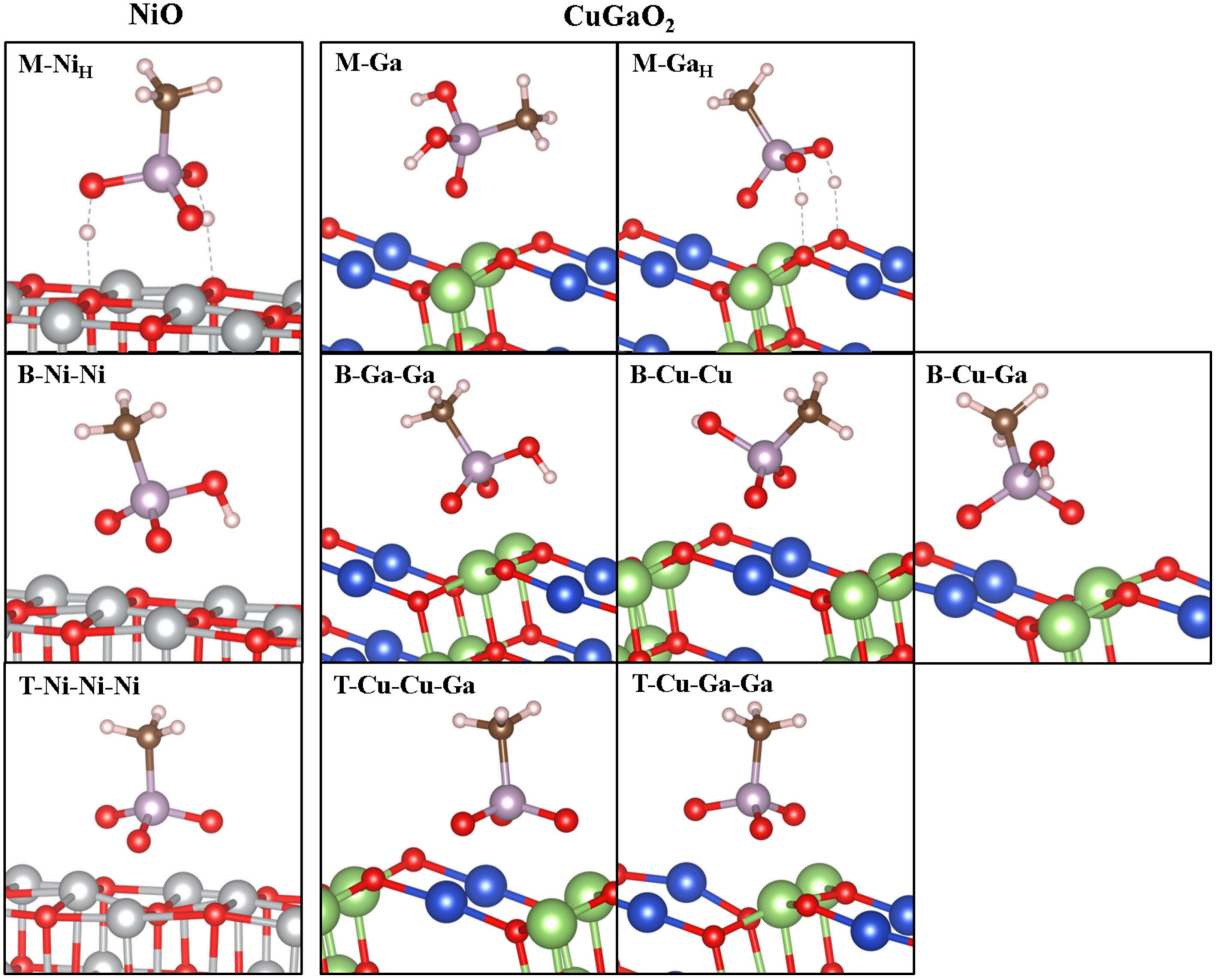
Optimized structures of CH_3_PO_3_H_2_ on CuGaO_2_ (011) 2 × 1 × 3 L slab (right) and comparison to NiO (001) (left). Labels according to the anchoring mode [monodentate (M), bidentate (B), and tridentate (T)] and to the surface atoms involved in the adsorption process. Sub index “H” indicates H bonding (dashed gray line) between the OH group and surface oxygen atom upon relaxation in M cases. Color legend: Ni (gray), Cu (blue), Ga (green), O (red), C (brown), H (light pink), and P (lavender).

Analogously, we have explored all possible binding modes of the phosphonic acid group on CuGaO_2_ (011). Relaxed geometries of CH_3_PO_3_H_2_ on CuGaO_2_ (011) in monodentate (M), bidentate (B), and tridentate (T) binding modes are shown in Figure 4, while corresponding structural parameters and E_ads_ are listed in Table 2 together with those of CH_3_PO_3_H_2_ on NiO (001).

**Table 2:** Selected structural parameters for relaxed CH_3_PO_3_H_2_ anchored on CuGaO_2_ (011) in comparison to those on NiO (001) and of the isolated molecule calculated at the DFT(PBE)+U level of theory in vacuum.

		**CH**_****3****_**PO**_****3****_**H**_****2****_ **Main structural parameters** **(Å)**										
	**Anchoring mode**	**d_**O1-P**_**	**d_**O2-P**_**	**d_**O3-P**_**	d$d_{O-H}^{*}$							**E_**ads**_ (eV)**
Isolated		1.48	1.61	1.62	0.98							
On NiO (001)						**d**_**O1-Ni**_		**d**_**O2-Ni**_		**d**_**O3-Ni**_	**d**_H-Os_^*^	
	M-Ni_H_	1.51	1.58	1.59	1.05	2.05		–		–	1.58	−1.08
	B-Ni-Ni	1.53	1.53	1.63	0.98	2.00		2.00		–	2.37	−1.18
	T-Ni-Ni-Ni	1.55	1.55	1.59	–	2.03		2.04		1.98	–	−1.08
On CuGaO_2_ (011)						**d**_**O1-Ga**_	**d**_**O1-Cu**_	**d**_**O2-Ga**_	**d**_**O2-Cu**_	**d**_**O3-Cu**_	**d**_H-Os_^*^	
	M-Ga	1.50	1.60	1.61	0.97	2.18	–	–	–	–	–	−0.13
	M-Ga_H_	1.53	1.58	1.58	1.04	2.03	–	–	–	–	1.60	−1.05
	B-Ga-Ga	1.53	1.54	1.61	0.99	2.00	–	2.02	–	–	2.13	0.03
	B-Cu-Cu	1.48	1.52	1.64	0.98	–	1.99	–	2.14	–	–	1.38
	B-Ga-Cu	1.50	1.55	1.64	0.98	1.99	–	–	1.91	–	–	0.55
	T-Ga-Ga-Cu	1.54	1.55	1.56	–	1.96	–	1.97	–	1.88	–	2.25
	T-Cu-Cu-Ga	1.50	1.55	1.57	–	–	1.91	1.92	–	1.84	–	3.03

Labels of anchoring modes as in Figure 4. O_1_ and O_2_/O_3_ correspond to phosphoryl and OH/OH oxygen atoms in isolated CH_3_PO_3_H_2_, respectively. O_s_ identifies a superficial oxygen atom. Values reported with an asterisk (*) correspond to the average value with a maximum standard deviation of 0.01 Å. Adsorption energies (E_ads_) in eV are collected in the last column. Bond lengths in the adsorbate (dX-C) and distance between the surface and the adsorbate (dCu-X, dGa-X, dNi-X, dOs-X) with X = O1, O2 or H.

* *) correspond to the average value with a maximum standard deviation of 0.01 Å. Adsorption energies (E_ads_) in eV are collected in the last column*.

In this case, only the monodentate adsorption modes on surface Ga atoms are favored. In particular, we find two stable M geometries, one without any H-bonding to the surface (M-Ga) and one where both the two OH groups form H-bonds with the delafossite surface O atoms (M-Ga_H_). Such H bonds stabilize the latter by 0.92 eV with respect to M-Ga. We explored different starting geometries with phosphoryl oxygen O_1_ linked to surface Cu but all evolve into either M-Ga or M-Ga_H_ upon relaxation. As in NiO, the –PO_3_H_2_ group in M binding (with H bonding) is more strongly bound than –COOH. As for –COOH, we find that only M binding of the –PO_3_H_2_ group is stable on the delafossite surface, differently from NiO, where M, B, and T modes are all strongly anchored to the surface with E_ads_ lying in a narrow window of energy. In CuGaO_2_, B modes present positive E_ads_ that go from the few eV of the B-Ga-Ga case to the 1.38 eV in the B-Cu-Cu case. In all the tridentate modes, adsorption energies are very high. So, we can conclude that –PO_3_H_2_ anchoring group will bind to CuGaO_2_ (001) preferentially through a M-Ga_H_-like anchoring mode, with small amounts of M-Ga-like and negligible presence of B-Ga-Ga.

In order to evaluate to what extent the selectivity toward monodentate binding on CuGaO_2_ for both anchoring groups described above is affected by Mg-doping and the presence of the solvent, we have also calculated E_ads_ of each anchoring group/mode in the pristine delafossite slab considering acetonitrile as solvent and on the slab containing 3.3% of Mg, both in vacuum and in acetonitrile. We chose acetonitrile as solvent since it is one of the most widely employed solvents in DSSCs and it was also employed as a solvent in the experimental electrolyte compositions we refer to for open circuit voltage determination.

These three additional sets of E_ads_, together with those calculated in vacuum on the pristine slabs are gathered in Table 3.

**Table 3:** Calculated adsorption energies (E_ads_) for CH_3_COOH and CH_3_PO_3_H_2_ on CuGaO_2_ (001) at the DFT(PBE)+U level of theory in vacuum and acetonitrile (implicit solvent), without and with Mg doping (3.3%).

**E**_****ads****_ **(eV)**					
**Anchoring group**	**Anchoring mode**	**CuGaO_**2**_ vacuum**	**Mg:CuGaO_**2**_ vacuum**	**CuGaO_**2**_ acetonitrile**	**Mg:CuGaO_**2**_ acetonitrile**
CH_3_COOH	M-Ga_H_	**−0.765**	**−1.150**	**−0.580**	**−0.776**
	M-Cu_H_	**−0.191**	**−0.663**	0.233	**-0.360**
	B-Ga-Ga	0.516	0.114	0.434	0.139
	B-Cu-Cu	1.597	1.741	1.926	0.357
	B-Cu-Ga	0.715	0.460	0.928	0.334
CH_3_PO_3_H_2_	M-Ga	**−0.128**	**−0.646**	0.160	**−0.121**
	M-Ga_H_	**−1.050**	**−1.638**	**−0.525**	**−1.121**
	B-Ga-Ga	0.026	0.077	0.041	0.019
	B-Cu-Cu	1.378	0.854	1.935	1.219
	B-Cu-Ga	0.548	0.307	0.547	0.333
	T-Ga-Ga-Cu	2.253	2.492	1.764	1.512
	T-Cu-Cu-Ga	3.030	2.546	2.656	1.756

**Figure 5:**
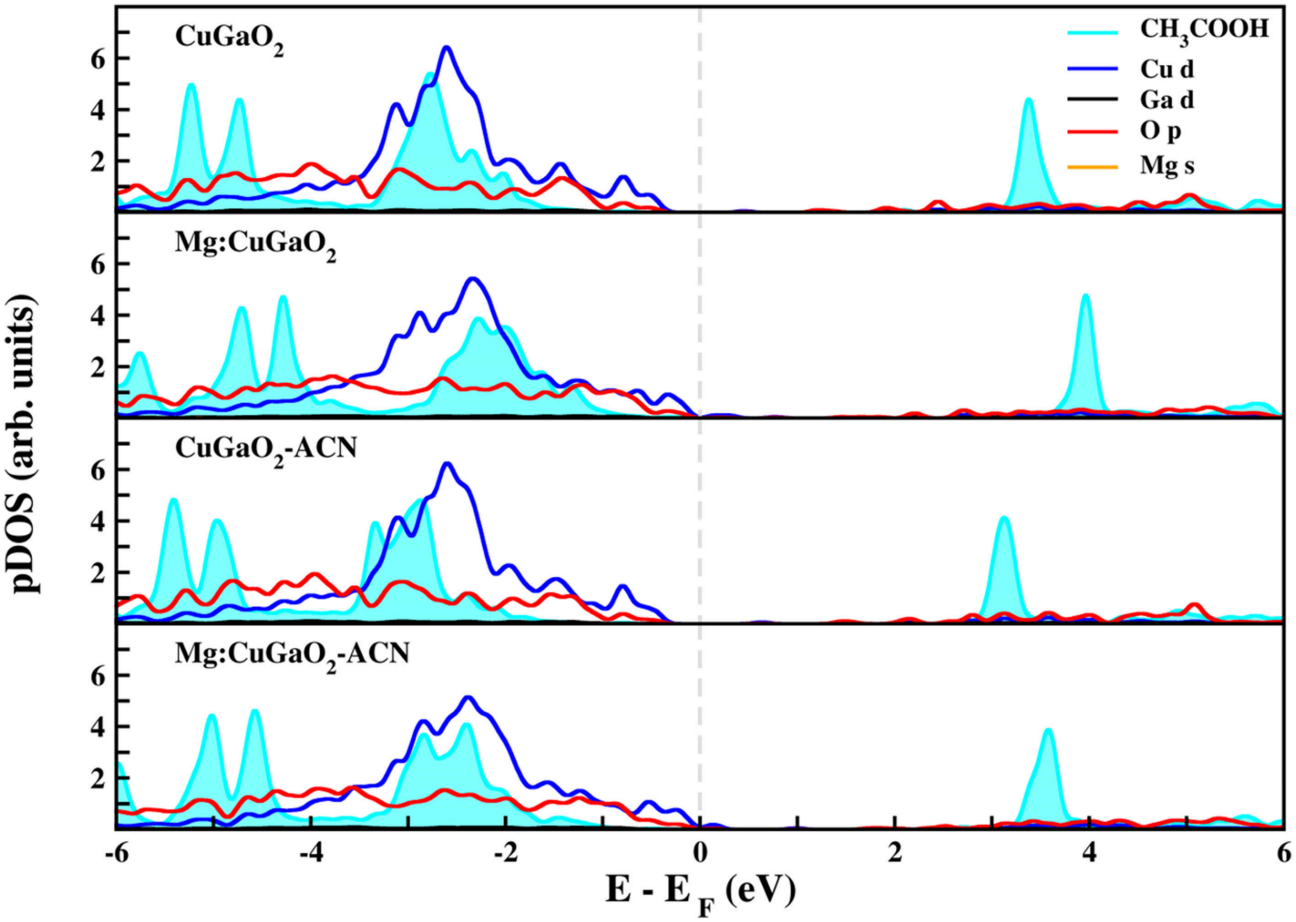
Projected density of states (pDOS) calculated at the DFT(PBE)+U level of theory of CH_3_COOH adsorbed on CuGaO_2_ (011). Fermi level (dashed gray line) has been set to zero.

*Anchoring mode labels as in Figures 4, 5. Negative binding energies are highlighted in bold*.

From our calculations, both anchoring groups bind significantly more strongly to the Mg-doped surface than on the undoped surface with E_ads_ that decrease almost uniformly around ~0.5 eV. There are very few exceptions where E_ads_ suffers a negligible increase (max. ~0.05 eV) upon Mg-doping as CH_3_COOH-B-Cu-Cu and CH_3_PO_3_H_2_-B-Ga-Ga/T-Ga-Ga-Cu. This stabilizing effect can be explained to the higher Lewis acidity of oxide surfaces upon p-type doping, where a hole has been introduced, increasing the ability of such oxide to accept electron-donating species (Metiu et al., 2012). We must note here that, as in bulk CuGaO_2_ (Schiavo et al., 2018), the presence of Mg does not lead to a significant localized oxidation of any particular cation but to the hole being distributed among all Cu atoms of the system (each Cu losing max. 0.08 e^−^), with even smaller involvement of Ga. This explains the uniform decrease in E_ads_ in all surface sites and not only on Cu sites. Regarding the solvent, as common trend to all the M and B anchoring modes, the dielectric continuum increases E_ads_ but not to the same extent. Only in the cases of T-modes in CH_3_PO_3_H_2_, it decreases by ~0.5 eV but this decrease is not enough to stabilize the very unfavorable T anchoring modes. From a general perspective, the acetonitrile solvent medium with a mild dielectric constant is expected to weaken the ionic contribution to the bonding between anchoring group O atoms and Ga surface sites. At the same time, for the T modes, the solvent is able to stabilize the dipole moments of the –OH surface species that are formed during the anchoring. In any case, the overall adsorption energy landscape is not qualitatively changed when both the electrolyte solvent and the Mg-dopant are taken into account. E_ads_ values are analogous to those calculated for the pristine slab in vacuum, as the two effects balance each other. Thus, we can state that M binding is likely the preferred anchoring mode for CH_3_COOH and CH_3_PO_3_H_2_ in operating conditions. This result is of utmost importance for the cell performance since it has been shown that the driving force for hole injection between the dye HOMO and the VB of the semiconductor is maximized in M binding modes where no H has been released to the surface and the interfacial dipole at the electrode surface is minimum (Muñoz-García and Pavone, 2015). Moreover, we can state that Mg doping, besides having an effect on improving p-type conductivity and nanoparticle morphology (Renaud et al., 2014), improves dye coverage by increasing the affinity between the delafossite and dye anchoring groups.

**Figure 6:**
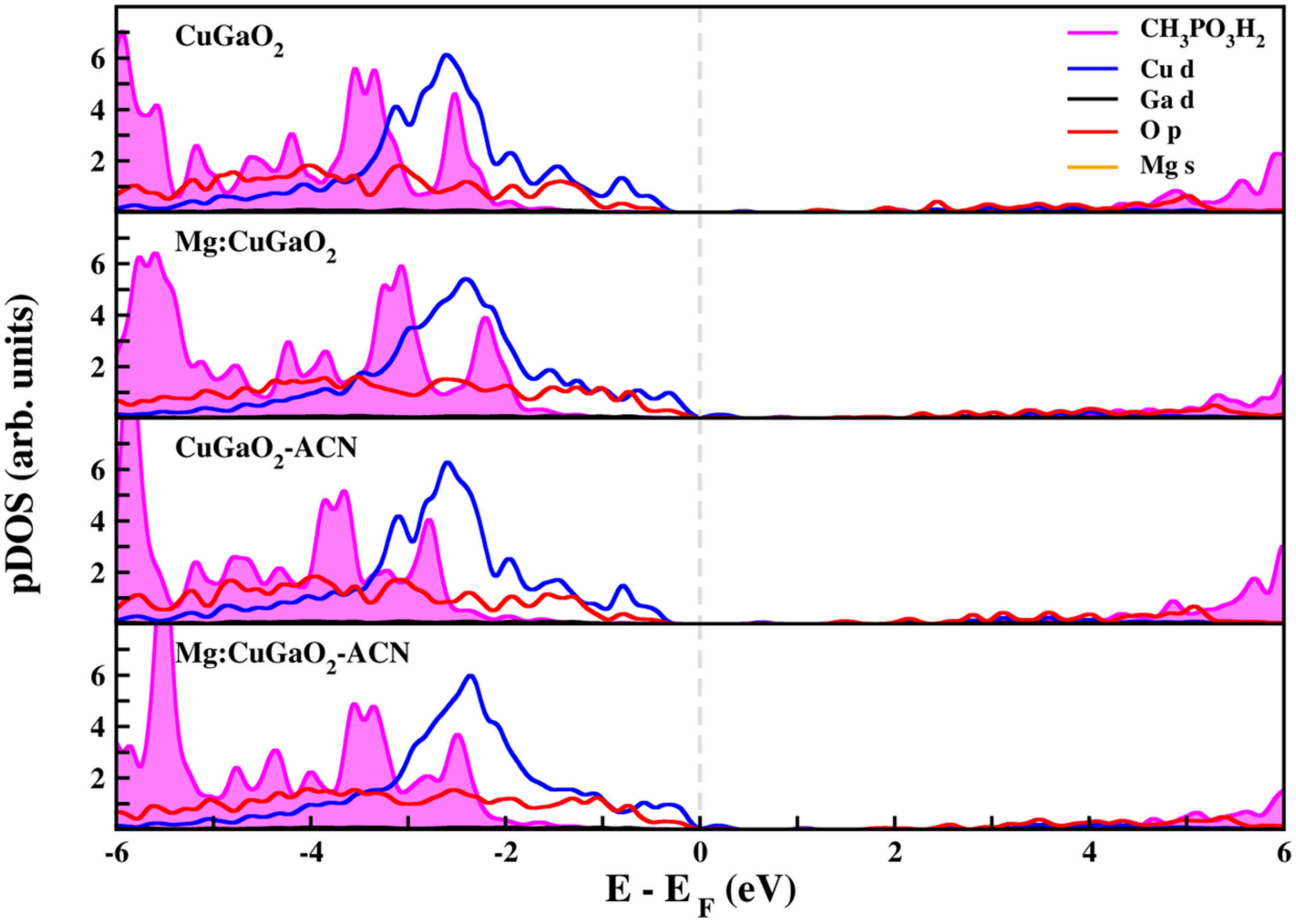
Projected density of states (pDOS) calculated at the DFT(PBE)+U level of theory of CH_3_PO_3_H_2_ adsorbed on CuGaO_2_ (011). Fermi level (dashed gray line) has been set to zero.

Figures 5, 6 show the projected density of states of CuGaO_2_(011) with anchored CH_3_COOH and CH_3_PO_3_H_2_, respectively, in the most stable binding mode (M-Ga_H_). We have considered all the four systems for which the adsorption energies were computed: pristine/vacuum, Mg-doped/vacuum, pristine/acetonitrile, and Mg-doped/acetonitrile. In all these cases, the molecular states of the anchoring groups are far from the semiconductor VB edge and, hence, they would not contribute to electron/hole transport process. These results are consistent with those recently computed for Ph-COOH and silanes on NiO (Wykes et al., 2016), in line with what is expected for the proper functioning of the solar cell.

## Conclusions

This work reports an *ab initio* study of CuGaO_2_ delafossite as alternative to NiO in p-type DSSCs. The semiconductive copper delafossite with chemical formula CuMO_2_ presents a lower absolute position of the valence band edge than NiO. Thus, besides providing a higher open circuit potential (V_OC_) when used with traditional I^−^/${\text{I}}_{3}^{-}$ than NiO, it enables the use of new and high performing Co- and Cu-based electrolytes. Gallium delafossite CuGaO_2_ is of particular interest since it can be easily doped with Mg, which enhances the p-type conductivity and the shape and morphology of delafossite nanoparticles. First, we have focused here on studying the change in the valence band absolute position after Mg doping: with ~3 and ~6% dopant contents the V_OC_ of the copper gallium delafossite is mostly unchanged, in agreement with experiments.

Due to the practical difficulties of sensitizing delafossite oxides, we have addressed the adsorption properties of two anchoring groups widely used for grafting dye molecules on semiconductor surfaces: –COOH and –PO_3_H_2_ (carboxylic acid and phosphonic acid, respectively) on the most stable CuGaO_2_ surface, i.e., the (011). We characterized the interaction between these anchoring groups and the surface in terms of anchoring mode minimum-energy structures and corresponding adsorption energies. We have dissected the effects of Mg doping and of the presence of the solvent on these features, as well as on the electronic structure, which is of key importance to DSSC operation. Contrary to what happens in the case of NiO, on delafossite surface there is a strong selectivity toward monodentate binding modes for both the carboxylic and phosphonic anchoring groups, with a particular affinity toward the Ga surface sites. Since it has been shown that driving force for hole injection from the dye to the semiconductor is jeopardized when protic groups release H to oxide surfaces in bidentate and tridentate modes, our results point out that the combination of –COOH or –PO_3_H_2_ anchoring groups with CuGaO_2_ might deliver much better performance than with NiO. Besides, our calculations show that Mg doping increases the affinity of CuGaO_2_ surface for both anchoring groups and, thus, the better performances of Mg-containing samples can be also ascribed to a higher sensitization driven by more favorable adsorption energies.

In conclusion, this work provides a first theoretical characterization of the interface between delafossite oxide and the –COOH or –PO_3_H_2_ anchoring groups, thus paving the route to further studies on full dye-sensitized delafossite-based photocathodes in order to help the development of p-type DSSC technologies with first-principles derived rational design guidelines.

## Data Availability

The datasets generated for this study are available on request to the corresponding author.

## Author Contributions

AM-G and MP designed the research. LC, ES, and CB performed the calculations. AM-G, PM, and MP rationalized the results. All authors contributed in writing and revising the manuscript.

### Conflict of Interest Statement

The authors declare that the research was conducted in the absence of any commercial or financial relationships that could be construed as a potential conflict of interest.
