# Supplementary material for: Ab initio Study of Anchoring Groups for CuGaO2 Delafossite-Based p-Type Dye Sensitized Solar Cells
Source: Front Chem. 2019 Mar 29;7:158. doi: 10.3389/fchem.2019.00158 (PMC6449920; doi:10.3389/fchem.2019.00158)
Supplement: Supplementary file 1 [file Table_1.DOCX]

***Supplementary material***

Structure coordinates files obtained from optimization calculations are reported in the following.

**
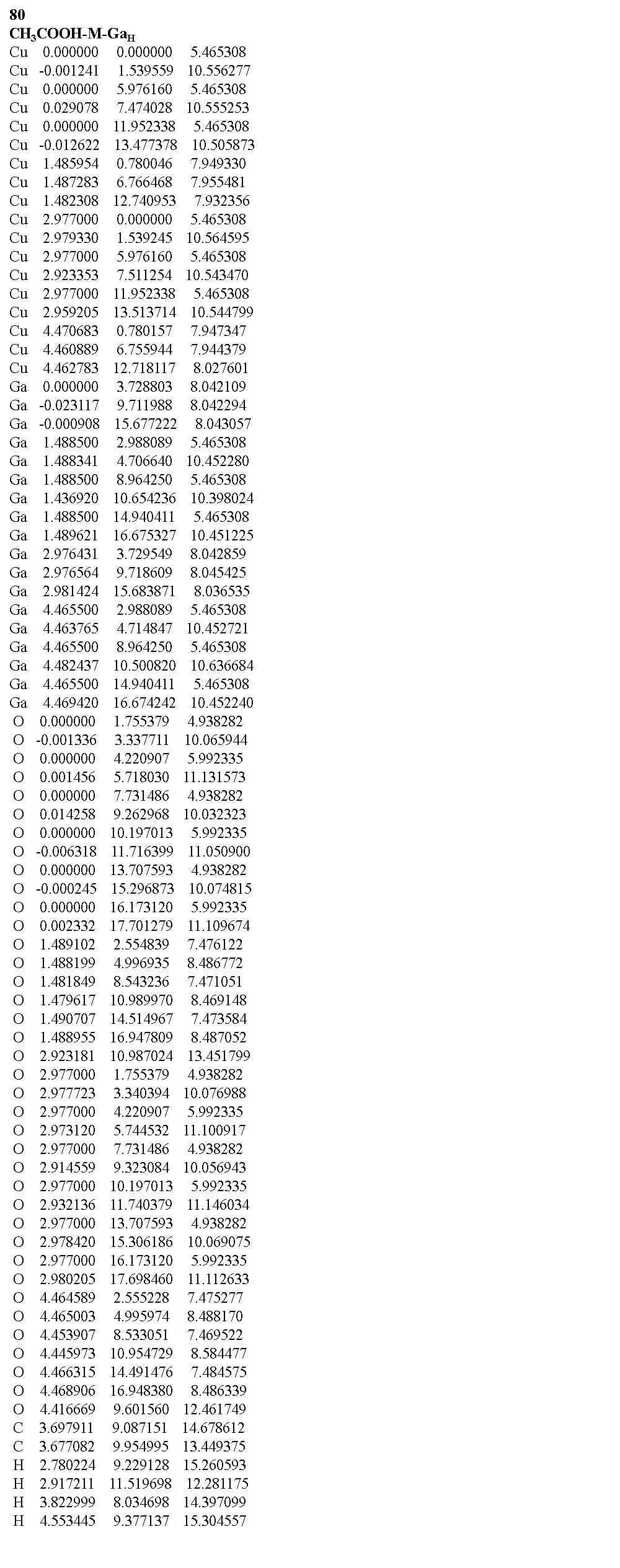
**

**Supplementary Figure 1** xyz coordinates obtained from optimization calculations performed for CH_3_COOH anchoring group in M-Ga_H_ adsorption mode.

**
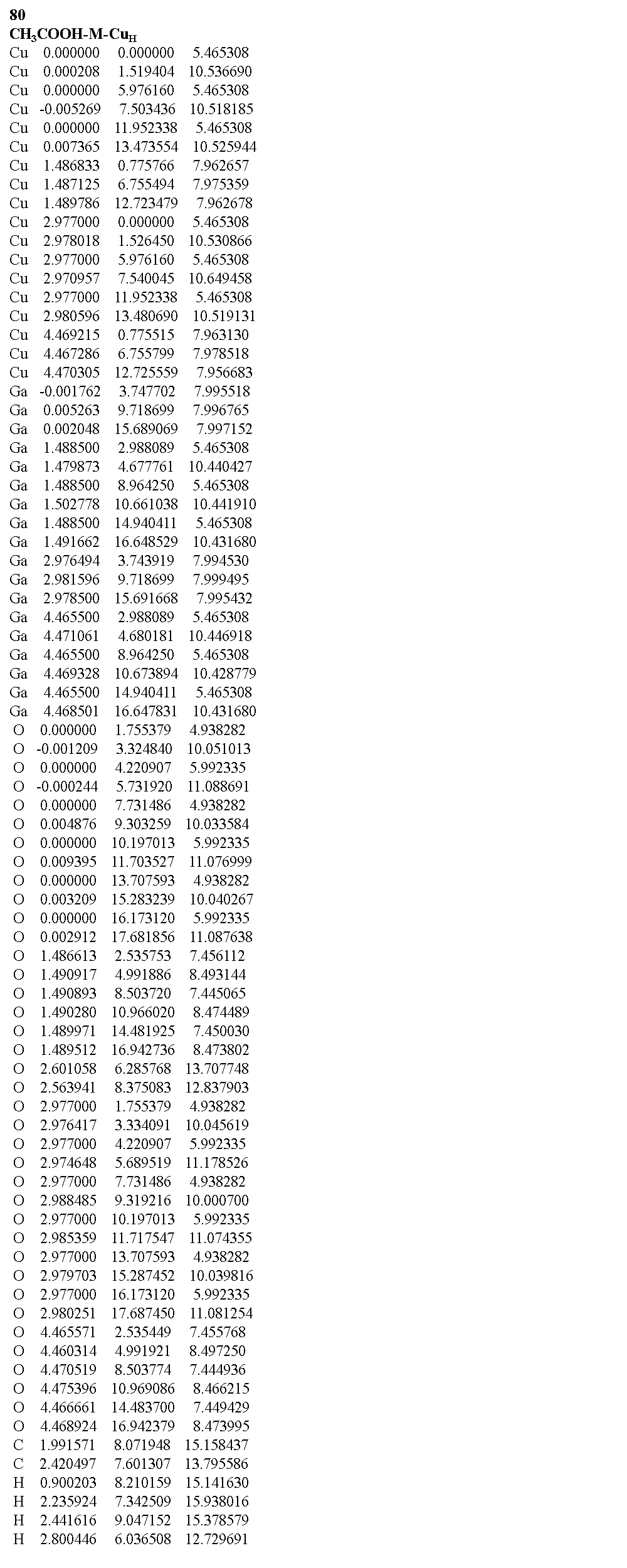
**

**Supplementary Figure 2** xyz coordinates obtained from optimization calculations performed for CH_3_COOH anchoring group in M-Cu_H_ adsorption mode.

**
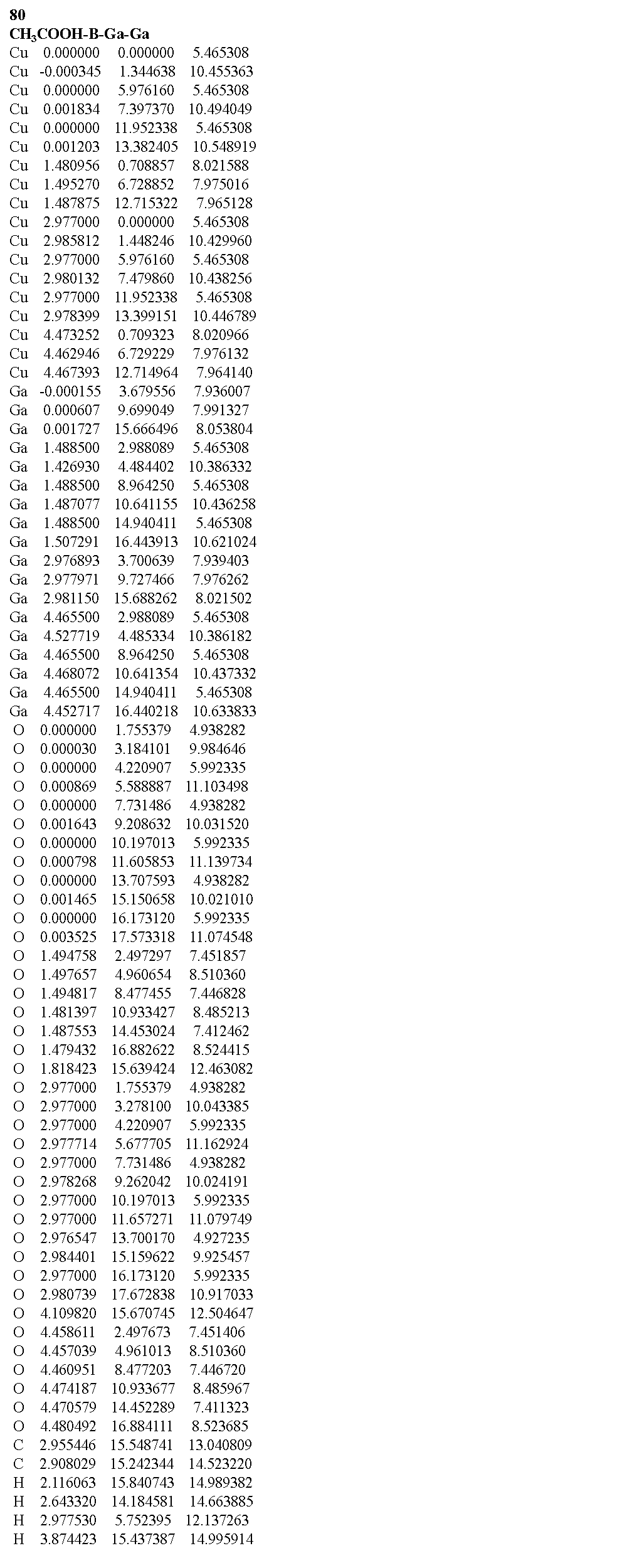
**

**Supplementary Figure 3** xyz coordinates obtained from optimization calculations performed for CH_3_COOH anchoring group in B-Ga-Ga adsorption mode.

**
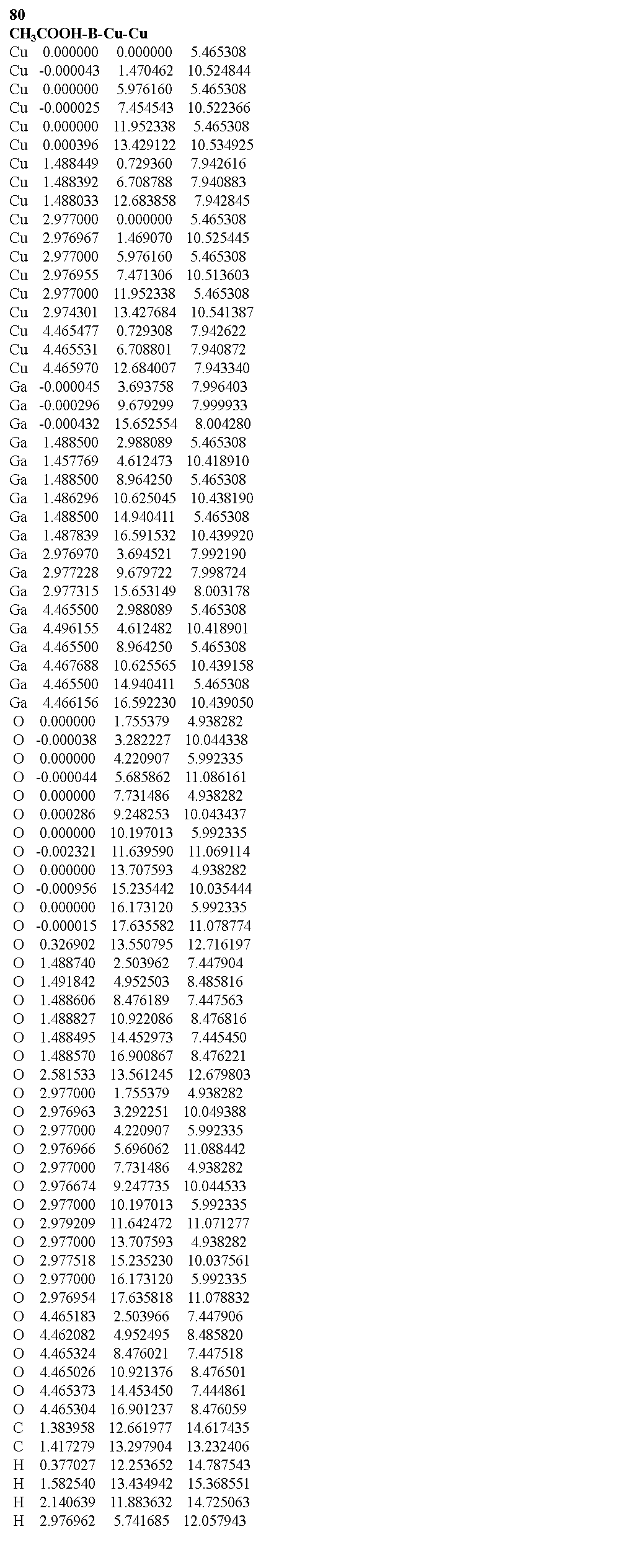
**

**Supplementary Figure 4** xyz coordinates obtained from optimization calculations performed for CH_3_COOH anchoring group in B-Cu-Cu adsorption mode.

**
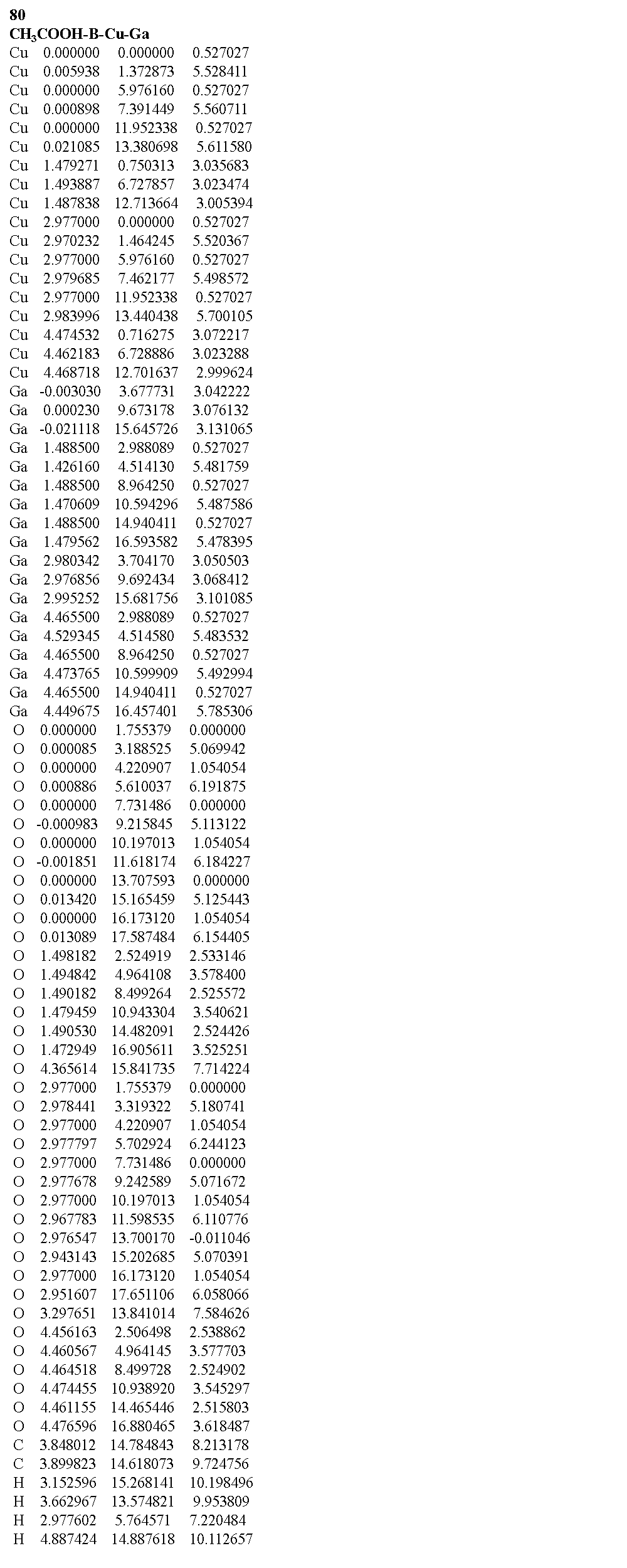
**

**Supplementary Figure 5** xyz coordinates obtained from optimization calculations performed for CH_3_COOH anchoring group in B-Cu-Ga adsorption mode.

**
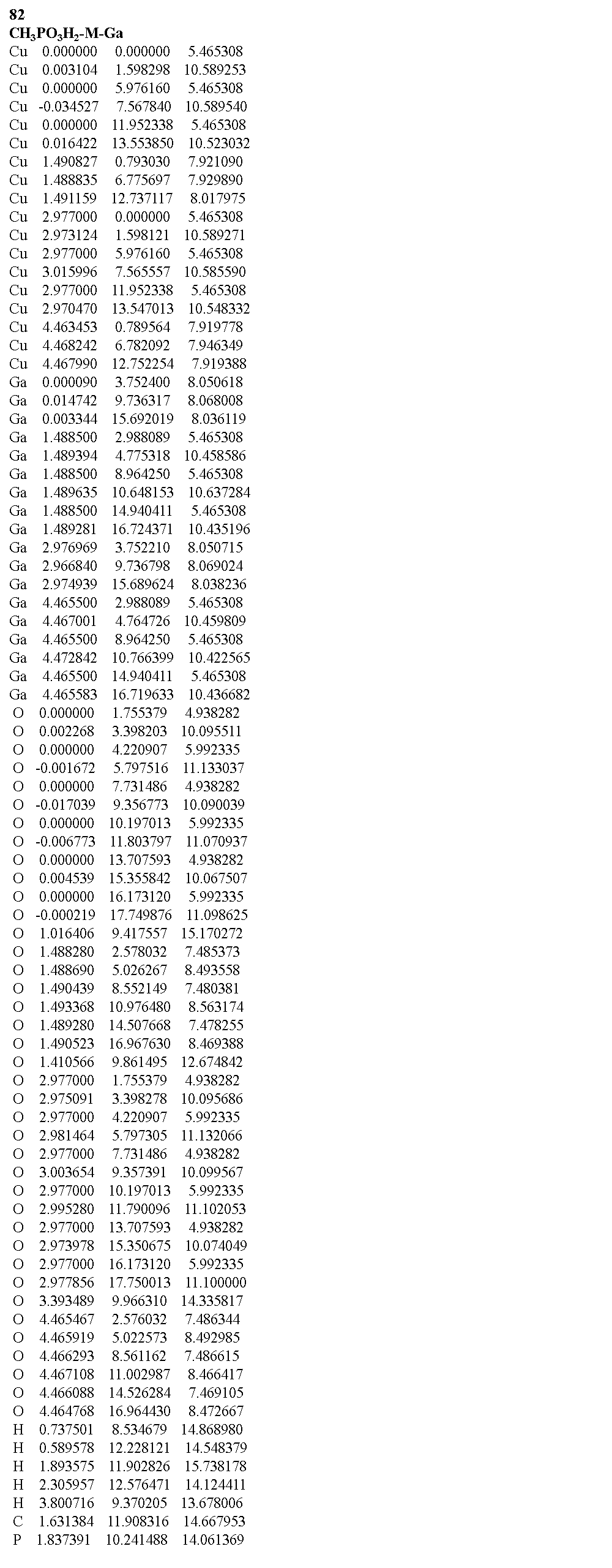
**

**Supplementary Figure 6** xyz coordinates obtained from optimization calculations performed for CH_3_PO_3_H_2_ anchoring group in M-Ga adsorption mode.

**
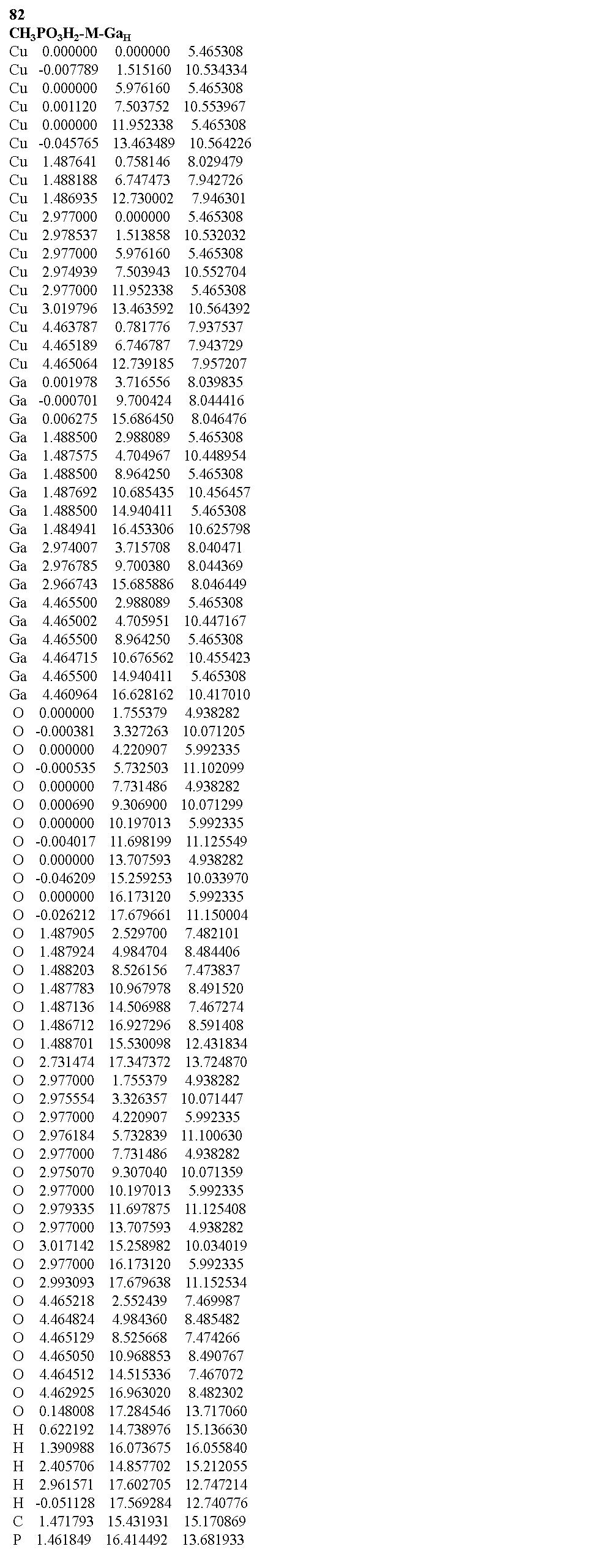
**

**Supplementary Figure 7** xyz coordinates obtained from optimization calculations performed for CH_3_PO_3_H_2_ anchoring group in M-Ga_H_ adsorption mode.

**
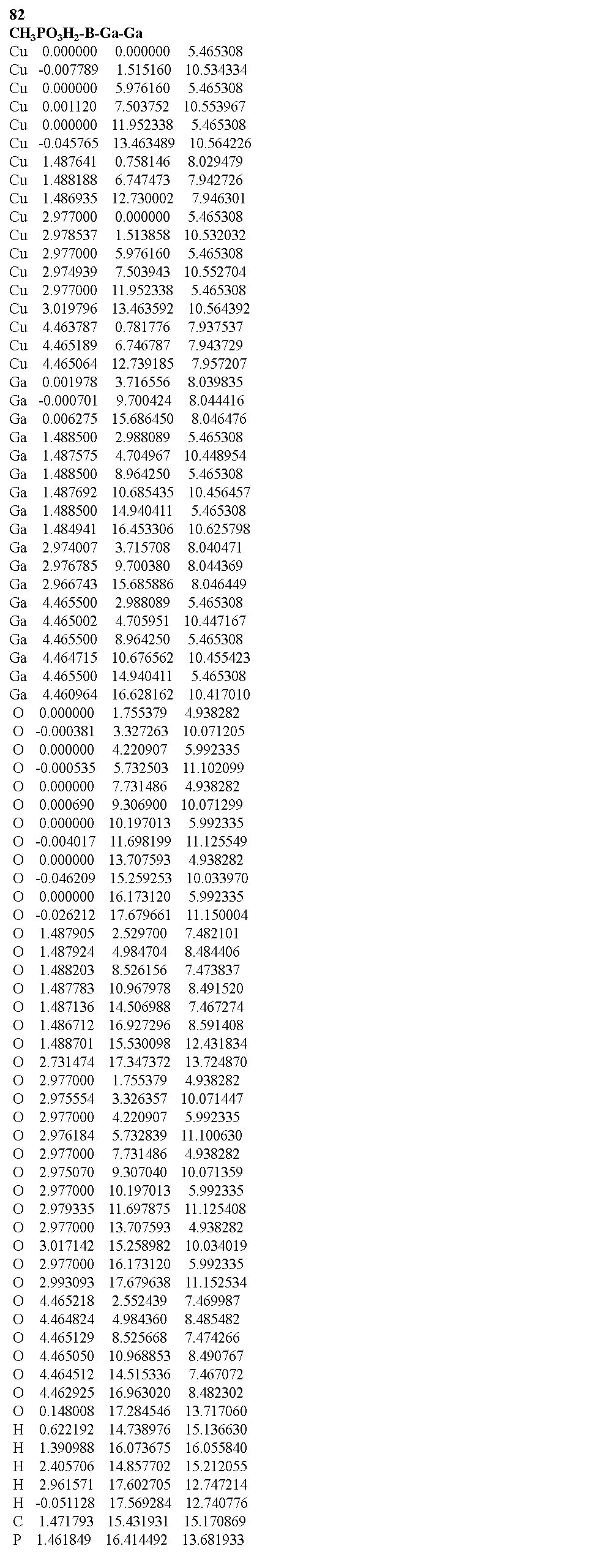
**

**Supplementary Figure 8** xyz coordinates obtained from optimization calculations performed for CH_3_PO_3_H_2_ anchoring group in B-Ga-Ga adsorption mode.

**
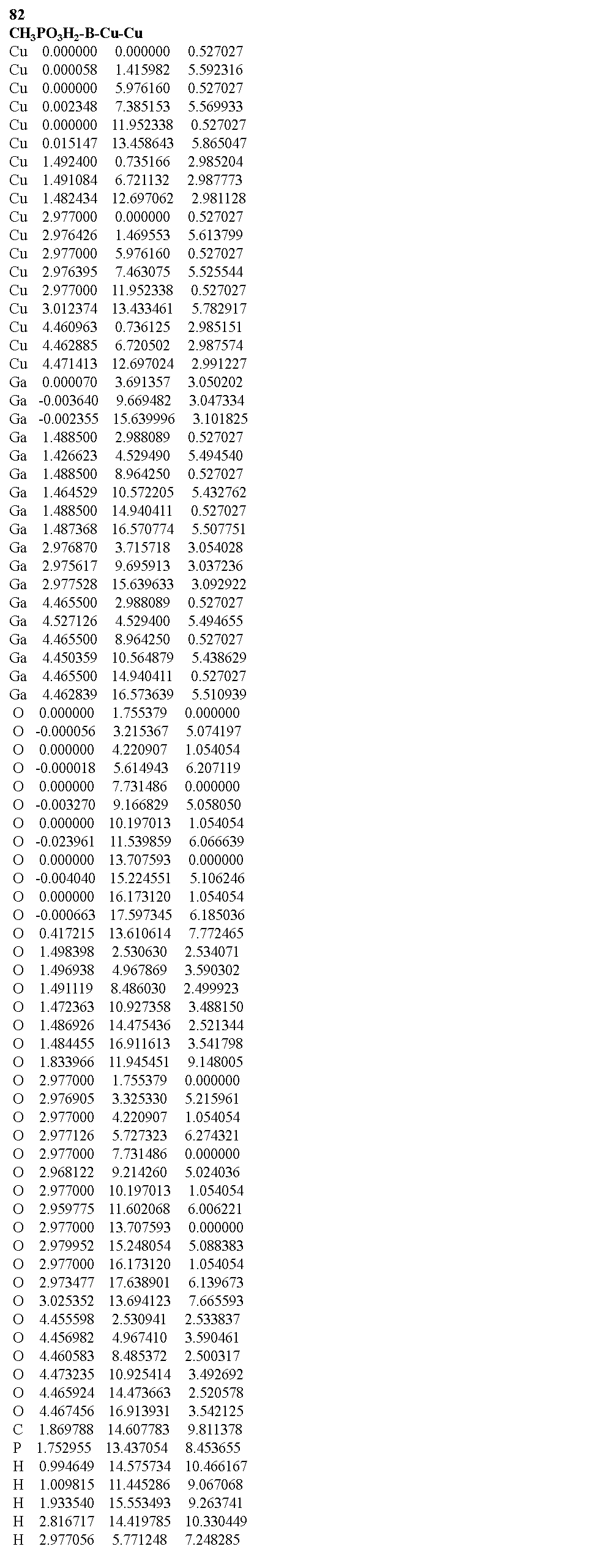
**

**Supplementary Figure 9** xyz coordinates obtained from optimization calculations performed for CH_3_PO_3_H_2_ anchoring group in B-Cu-Cu adsorption mode.

**
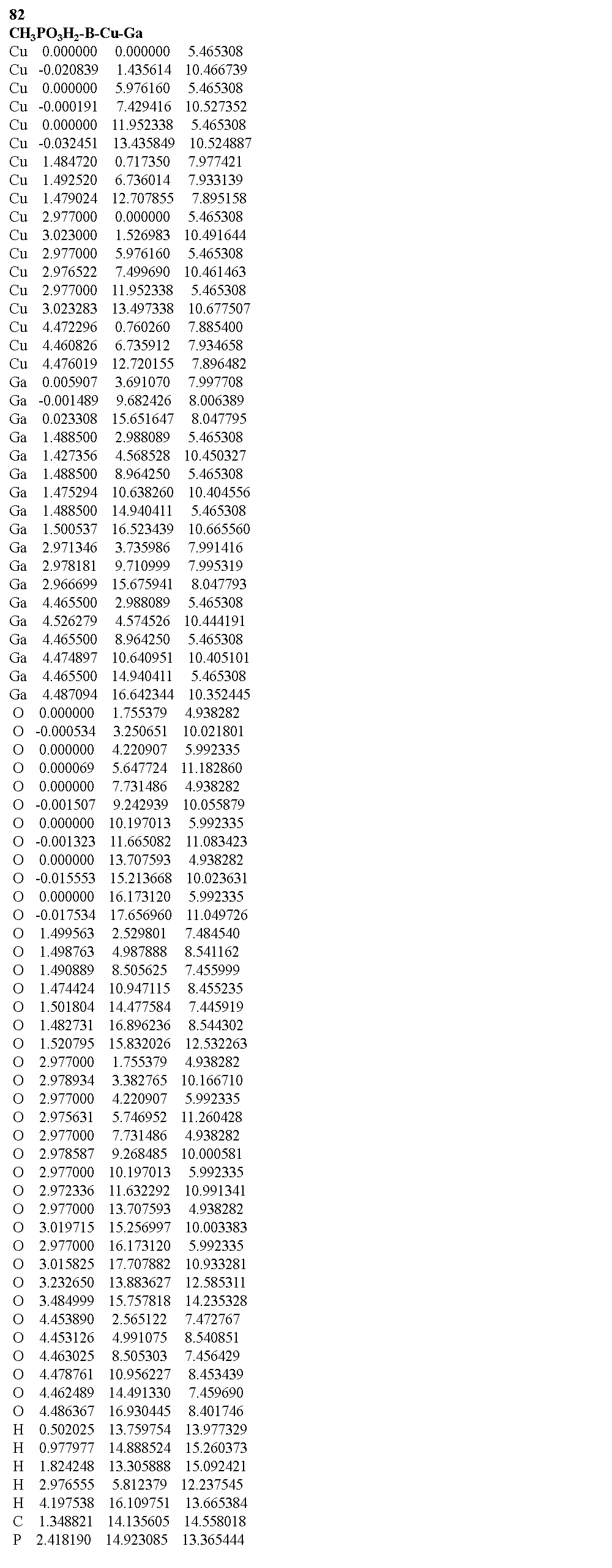
**

**Supplementary Figure 10** xyz coordinates obtained from optimization calculations performed for CH_3_PO_3_H_2_ anchoring group in B-Cu-Ga adsorption mode.

**
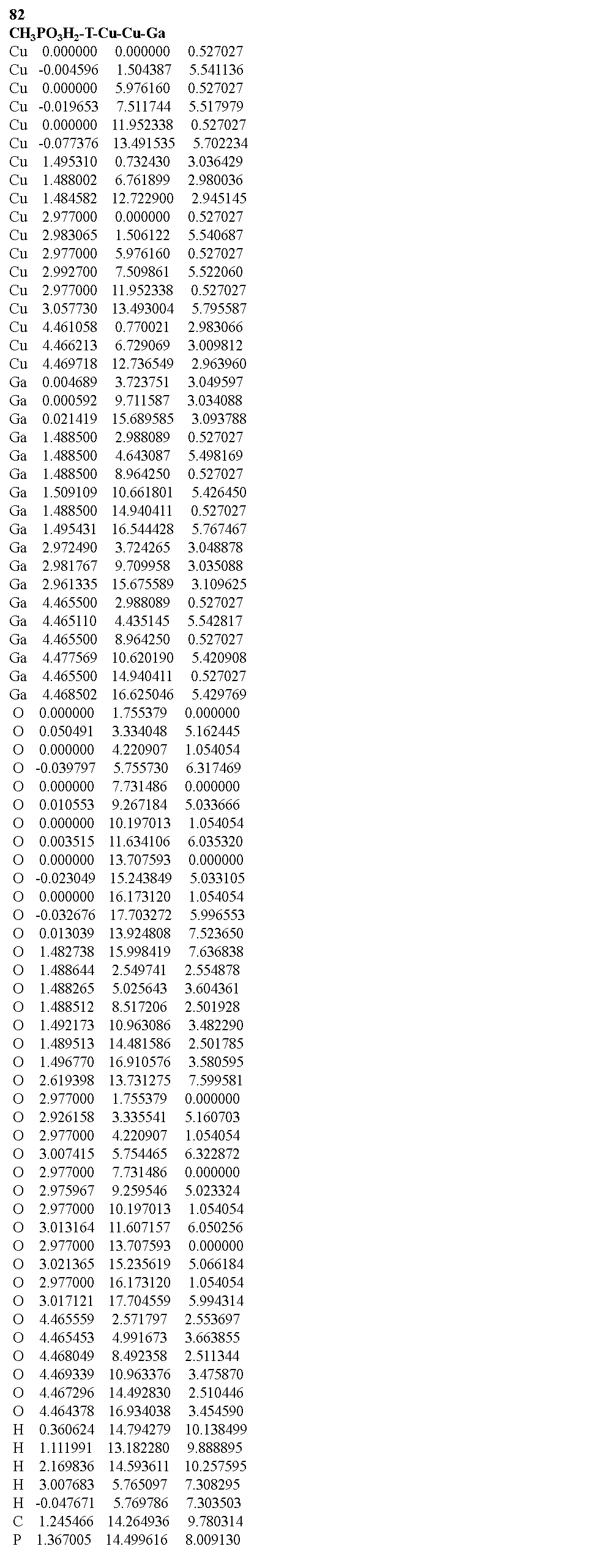
**

**Supplementary Figure 11** xyz coordinates obtained from optimization calculations performed for CH_3_PO_3_H_2_ anchoring group in T-Cu-Cu-Ga adsorption mode.

**
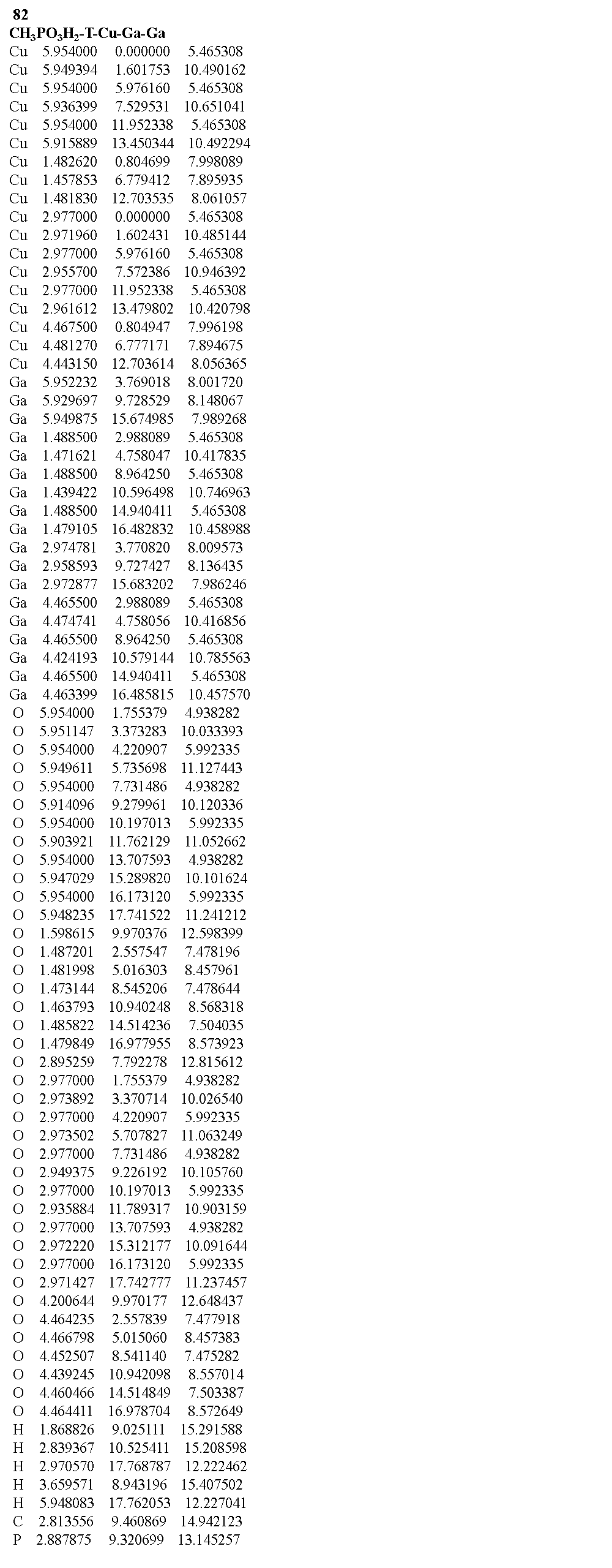
**

**Supplementary Figure 12** xyz coordinates obtained from optimization calculations performed for CH_3_PO_3_H_2_ anchoring group in T-Cu-Ga-Ga adsorption mode.
